# ‘A troublesome girl is pushed through’: Morality, biological determinism, resistance, resilience, and the Canadian child migration schemes, 1883–1939

**DOI:** 10.1177/09526951211036553

**Published:** 2021-09-14

**Authors:** Wendy Sims-Schouten

**Affiliations:** University of Portsmouth, UK

**Keywords:** biological determinism, child migration schemes, critical realism, morality, resilience

## Abstract

This article critically analyses correspondence and decisions regarding children/young people who were included in the Canadian child migration schemes that ran between 1883 and 1939, and those who were deemed ‘undeserving’ and outside the scope of the schemes. Drawing on critical realist ontology, a metatheory that centralises the causal non-linear dynamics and generative mechanisms in the individual, the cultural sphere, and wider society, the research starts from the premise that the principle of ‘less or more eligibility’ lies at the heart of the British welfare system, both now and historically. Through analysing case files and correspondence relating to children sent to Canada via the Waifs and Strays Society and Fegan Homes, I shed light on the complex interplay between morality, biological determinism, resistance, and resilience in decisions around which children should be included or excluded. I argue that it was the complex interplay and nuance between the moral/immoral, desirable/undesirable, degenerate, and capable/incapable child that guided practice with vulnerable children in the late 1800s. In judgements around ‘deservedness’, related stigmas around poverty and ‘bad’ behaviour were rife. Within this, the child was punished for his/her ‘immoral tendencies’ and ‘inherited traits’, with little regard for the underlying reasons (e.g. abuse and neglect) for their (abnormal) behaviour and ‘mental deficiencies’.

## Introduction

Between 1869 and 1939, over 100,000 children and young people (aged between 5 and 14 to start with, and largely aged 14–16 from 1909 onwards) were sent to Canada from the British Isles as part of the child emigration movement (Constantine, 2002; Jenkins, 2000). Motivated by social and economic forces, the child migration schemes were heralded for providing pauper children with a better chance for a healthy, moral life in rural Canada, where families welcomed them as a source of cheap farm labour and domestic help (Lynch, 2016; Parr, 1982). The child migration schemes were run by philanthropic agencies. Two such voluntary institutions were Fegan Homes and the Waifs and Strays Society; the former was responsible for sending 3200 boys to Canada between 1884 and 1915, and the latter for sending approximately 3500 children (both boys and girls) to Canada between 1883 and 1937 (*The J. W. C. Fegan British Home Children Newsletter Collection 1877 to 1920*, 2013; Kohli, 2003). Through analysing case files and correspondence relating to children sent to Canada via the Waifs and Strays Society and Fegan Homes between 1883 and 1939, I aim to shed light on the complex interplay between morality, biological determinism, and resilience in decisions around which children should be included or excluded. Specifically, I will show how child migration schemes were presented as moral programmes to ‘rescue’ children facing poverty or danger, on the one hand, while adopting discriminatory selection procedures framed within stereotypical judgements regarding ‘bad behaviour’ and mental inferiority, on the other. Here, the ‘rescued’ child/young person was positioned within a lower class/hierarchy and conceived as less worthy/able than other children (Sohasky, 2015). Fuelled by biological determinism and eugenics, the assumption was that a child's moral character was irretrievably shaped by heredity, resulting in child migrants being condemned as degenerate ‘slum kids’ (Buss, 1976; Partridge, 1912; Stewart, 2009). Between these positions was a tentative construction of some children as ‘capable’ and ‘resilient’, namely those who showed strength of character and an ability to change (Lynch, 2014; Parr, 1982). Yet other coping mechanisms, such as resistance to emigration and running away, were blamed on their upbringing and hereditary tendencies, leading to them being classed as ‘troublesome’ and hard to manage and placing them in the ‘undeserving’ category (Moss, Wildman, and Lamont, 2020; Sims-Schouten, 2020; Sims-Schouten, Skinner, and Rivett, 2019). I argue that the legacy of this complex interplay and nuance between the moral/immoral, desirable/undesirable, degenerate, and capable/incapable child that guided practice with vulnerable children in the late 1800s can still be seen in safeguarding and mental health support decisions today (Sims-Schouten, 2020; Sims-Schouten, Skinner, and Rivett, 2019).

While some writings highlight the altruistic motives of the voluntary agencies that sent children abroad – in her book on the ‘Middlemore Experience’, for instance, Roberts-Pichette (2016) constructs the child migration scheme in terms of an initiative that helped vulnerable children thrive – cases of abuse and neglect have also been widely reported in relation to the Canadian (as well as the Australian) child migration schemes (see Constantine, 1991; Independent Inquiry Into Child Sexual Abuse, 2018; Lynch, 2014). The selection process associated with UK child migration schemes to Canada located the child within a framework of both morality (i.e. the focus on rescuing the child from moral danger) and biological determinism (excluding ‘degenerate’ children and children with undesirable traits; Faulkner, 2011; Sims-Schouten, 2020; Swain and Hillel, 2010). Between these positions, there is also a sense of framing of particular kinds of child who were constructed as either capable of change, with strength of character and positive traits, or as hard to manage and difficult (Lynch, 2016; Moss, Wildman, and Lamont, 2020; Parr, 1982). The behaviour of the latter was blamed on their upbringing, rather than the upheaval of emigration, but could also be viewed as a strategy of resistance by children who had few means and methods of recourse (Moss, Wildman, and Lamont, 2020). Both framings, namely that of the child ‘capable of change’ and that of the ‘hard to manage child’, may have been early signs of reflecting what is now referred to as ‘resilience’. Although the term *resilience* was not used in relation to human behaviour and capability until the 1970s (Werner's research with deprived children in Hawaii is believed to be one of the first published studies with a focus on resilience; see Werner and Smith, 1977, 1982), there are examples of associations with resilience in earlier publications. An example is the work of Scottish author and government reformer Samuel Smiles, who published a book aptly titled Self*-Help* in 1859 and another entitled *Character* in 1871. In both books, Smiles referred to and championed the influence of character, courage, self-control, home power, and temper, all terms that are used in current research around resilience in children (e.g. Luthar, Cicchetti, and Becker, 2000; Sims-Schouten and Edwards, 2016). While some children and young people who were included in the child migration schemes on the surface appeared to adjust and showed ‘strength of character’ in light of the challenges faced, other children expressed resistance towards emigration by running away and being hard to manage (Moss *et al.*, 2017). ‘Strength of character’, compliance, and resistance could be explained and analysed in light of ‘self-help’, ‘character’, and ‘resilience’ (Smiles, 1859, 1871; Ungar, 2002, 2004) and can be used as tools for understanding the outcomes of the processes and use of decision-making by child rescue charities and schemes.

In light of constructions around morality, biological determinism, and resilience/resistance, this article critically analyses correspondence and decisions regarding children/young people who were included in the child migration schemes and those who were deemed ‘undeserving’ and outside the scope of the schemes. Drawing on critical realist ontology, a metatheory that centralises the causal non-linear dynamics and generative mechanisms in the individual, the cultural sphere, and wider society, the study starts from the premise that the principle of ‘less or more eligibility’ lies at the heart of the British welfare system, both now and historically (Bhaskar, 1989, 2014; Sims-Schouten, Riley, and Willig, 2007). Critical realism provides insight into oppression, inequality, and uneven practices through the search for generative mechanisms and causal factors that, combined, may have created a phenomenon over time and thus influenced particular outcomes and practices (Mutch, 2014; Sims-Schouten, Skinner, and Rivett, 2019; Wilson, 2020). As a result, it stimulates the drive to gain insight into the quandary between three structural concepts (Chauhan and Foster, 2014): ‘absence’ (under-representation, underprivilege, and what is missing in a context or institution/organisation, highlighting a possible need for a critical focus), ‘difference’ (stigmatic labelling, e.g. in relation to poverty, character, or self-control), and ‘threat’ (e.g. ‘immoral behaviour’ or ‘undesirable traits’). Critical realism can thus form the basis for research with a focus on making sense of child protection practices, taking account of the fact that these practices and related perceptions are both socially constructed and influenced by external factors and forces that can be real and independent of any one person or social group (Sayer, 2000; Sims-Schouten, Skinner, and Rivett, 2019). Historical investigations can explain some of the mechanisms at play at the field level, influencing particular (uneven) outcomes and practices, such as the legacy of the punitive ‘deserving/undeserving’ paradigm inherited from the New Poor Law of 1834 (Mutch, 2014; Sims-Schouten, 2020; Sims-Schouten and Riley, 2018). The New Poor Law was implemented to reduce spending on the poor by centralising the notion of eligibility, the idea that some people (e.g. the elderly, the infirm, widows) were deserving of welfare support due to an inability to work, through no fault of their own (Atherton, 2011; King, 2019; Royden, 2017). The ‘deserving/undeserving’ paradigm also played a significant role in decisions around which children should and should not be supported; its legacy can also be seen to have influenced the child migration schemes (Sims-Schouten, 2020)*.* For example, Lynch (2014) refers to the moral nature of the various child migration and child rescue schemes and highlights that within this certain children were perceived as outside the scope of the moral rescue scheme. The next section sheds light on the child migration schemes associated with Fegan Homes and the Waifs and Strays Society, which is followed by an analysis of the selection processes and framings (morality, biological determinism, resilience) of children included in the schemes.

## Child migration: Fegan Homes and the Waifs and Strays Society

The latter half of the 19th century saw the rise of the child rescue movement and philanthropic voluntary agencies providing institutional care and support for the poor, destitute, and orphaned young (Sims-Schouten, 2020; Skinner and Thomas, 2018). By the mid to late 1800s, there were a multitude of institutions in Britain that were used as a substitute for children's ‘natural’ homes, from orphanages (although it should be noted that these institutions also catered for children who were not orphans) to a wide range of other establishments run by charities, religious groups, workhouse authorities, local councils, and single individuals, serving particular purposes, such as moral protection and penal confinement (Higginbotham, 2017; King, 2003; Skinner and Thomas, 2018). At the same time, initiated by religious and charitable organisations, the child migration movement started to take off. One of the earliest of these organisations was the Children's Friend Society, established in 1830, which sent out its first party of child migrants to Australia in 1832 (Bagnell, 2001; Honeyman, 2012). In 1850, Parliament granted Poor Law guardians the ability to fund the emigration of children to the colonies. Between 1869 and the 1930s, over 100,000 child emigrants ended up in Canada alone as part of the child migration schemes facilitated by religious and charitable organisations (Kohli, 2003). Foregrounding the voluntary nature of the migration schemes and placing responsibility with the philanthropic institutions allowed the British government to give tacit support for child migration ‘at one remove’, without incurring the censure of powerful interest groups who opposed child migration (Grier, 2002). The Independent Inquiry Into Child Sexual Abuse (IICSA), a body tasked with investigating and reporting on the historical child migration schemes to Canada, Australia, New Zealand, and Southern Rhodesia, has observed that child migration was never entirely uncontroversial: reports as far back as the 1800s expressed significant criticisms of it (Independent Inquiry Into Child Sexual Abuse, 2018). Yet politics and economic benefits were consistently prioritised over the welfare of children.

This study draws on archival data (including correspondence, case files, emigration paperwork, reports, and magazines) associated with children sent to Canada by the Waifs and Strays Society and Fegan Homes. By analysing archival materials from Canada alongside those from the UK, I aim to shed light on emigration decisions and justifications from philanthropic institutions in the UK (namely the Waifs and Strays Society). I also examine correspondence from children prior to their move to Canada, as well as emigration paperwork, letters, decisions, and correspondence (relating to the Waifs and Stays Society and Fegan Homes) after their emigration to Canada. Here, I am specifically interested in language around behaviour, mental state, and deficiency, neglect, and character. Firstly, I accessed a total of 100 case files (consisting of correspondence from custodians, educators, medical officers, church reverends, and practitioners linked to asylums and industrial schools, as well as parents and children) at the archives of the Children's Society in London (formerly known as the Waifs and Strays Society). Only case files that referred to ‘Canada’ were selected; a search of the archives revealed a total of 1354 references to Canada. The search was narrowed down through the use of the keywords ‘behaviour’, ‘mental state’, ‘deficiency’, ‘neglect’, and ‘character’, resulting in a total of 100 case files. Secondly, I accessed 42 microfilm reels (consisting of roughly 1500 images each) from Library and Archives Canada (LAC) in Ottawa, comprising documents (minute books, emigration papers, and correspondence between receiving and sending homes) associated with children sent to Canada by two institutions: Fegan Homes (Volumes 1–3, 7, 8) and the Waifs and Strays Society (A-1137 through to A-1175). As with the search in the Children's Society archives in London, the following keywords were used when examining both the Waifs and Strays Society and Fegan Homes archives: ‘behaviour’, ‘mental state’, ‘deficiency’, ‘neglect’, and ‘character’. Here it needs to be acknowledged that work in archives is susceptible to chance and serendipity, and while the research is directed by the aims of the study and related keywords, the nature of the sources may either constrain the answers to the questions or suggest new directions, and data may have to be recoded to answer a new question (Mutch, 2014; Ventresca and Moor, 2002). Moreover, the timespan and archival data accessed (in this case, data linked to the Waifs and Strays Society and Fegan Homes) may offer only partial evidence for an interpretation (considering the range of different philanthropic institutions involved in the child migration movement). In her study of the Middlemore Homes, for example, Roberts-Pichette (2016) comes to the conclusion that the schemes led by the Middlemore Homes helped vulnerable children thrive. In my archival search, I used a number of keywords that were informed by the aims of the study, and I also narrowed down my search and recoded the findings, and was lucky enough to be able to consult with and draw on the expertise of archive staff (both at the Children's Society Archives and at LAC) who had a clear understanding of the collections, unprocessed materials, and related materials (Duff and Johnson, 2003). It was through the latter that I was introduced to the Fegan Homes archives, which were not digitised and required specific permission for viewing.

Fegan Homes was established by James W. C. Fegan in 1870 and catered for street boys (first in London and from 1872 further afield) and over the years opened a number of homes, missions, orphanages, schools, and training farms in London and elsewhere in England, including Ramsgate, Stony Stratford, Southwark, and Goudhurst (Fullerton, 1931). From 1884, Fegan started to send boys to Canada and opened distributing homes in Manitoba, Toronto, and Ontario (Kohli, 2003; Parker, 2010). Roughly 3200 Fegan boys ended up in Canada, between 1884 and 1915 and from the end of the First World War until 1939; most of the boys were placed on farms. The Waifs and Strays Society was established in 1881 by Edward Rudolph, with the goal of setting up homes for destitute children in connection with the Church of England, which, as far as possible, would provide children with a family environment rather than an institutional one (Higginbotham, 2017; Skinner and Thomas, 2018). Over 20,000 children from across England and Wales were cared for by the Waifs and Strays Society between 1881 and the end of the First World War. Between 1883 and 1937, the Waifs and Strays Society sent approximately 3500 children to Canada from its residential children's homes in England and Wales. During the period that the Waifs and Strays Society was active in Canada, it maintained six receiving homes: Gibbs’ Home in Sherbrooke, Quebec (girls’ home, 1884–97; boys’ home, 1897–1933); Benyon Home in Sherbrooke, Quebec (boys’ home, 1884–97); Our Western Home in Niagara-on-the-Lake, Ontario (girls’ home, 1897–1921); Elizabeth Rye Home in Toronto, Ontario (girls’ home, 1924–32); and Winnipeg Babies’ Home in Winnipeg, Manitoba (home for boys and girls aged 0–5, 1909–11; Parker, 2010).^1^ Until 1909, the children sent to Canada were aged between 5 and 14 and were mostly girls; boys tended to be sent mostly at age 14. In 1909, girls gained parity with boys when the Society increased their lower age limit to 14 years. In 1925, the age limit for girls was increased to 16 (Kohli, 2003).

Both Fegan Homes and the Waifs and Strays Society maintained a strict policy throughout the whole period they were involved in child emigration of requiring the consent of a parent or guardian to be given prior to a child being emigrated to Canada. Nevertheless, there is evidence that children were sent to Canada without parental consent, and it is not possible to ascertain whether children without parents or a guardian were more likely to be proposed for emigration than other children (Bagnell, 2001; Parker, 2010). The majority of the children sent to the receiving homes in Canada were eventually outsourced to work – boys on the farms and girls in domestic service. Many believed that these children would have a better chance for a healthy, moral life in rural Canada. Yet there is evidence that receiving households were often motivated more by the economic benefits that children of different ages could bring than by the symbolic ideal of supporting the civic and moral formation of a vulnerable child (Lynch, 2014). This is evident from patterns of children's movement between different households, which were often closely related to changes in the economic terms of their placement (Parr, 1994). Some households preferred to receive younger children, for whom they received regular boarding-out payment, and asked to return these children to organisational homes when they reached an age when such payments were no longer due (ibid.).

The child migration movement embodied a patchwork of practice grounded in notions of morality, the ‘deserving/undeserving’ paradigm, and biological determinism (Constantine, 2013; Delap, 2015; Sims-Schouten, 2020). The New Poor Law was implemented to reduce spending on the poor by centralising the notion of ‘deservedness’ and eligibility for support grounded in subjective judgements relating to people's ability and willingness to work and better themselves (Atherton, 2011; King, 2019; Royden, 2017; Sales, 2002). This also involved the positioning of children informed by deterministic assumptions associated with biological determinism, namely that a child's moral character was irretrievably shaped by heredity, as well as the assumption that children should take personal responsibility for their social condition as much as adults, in line with the ‘deserving/undeserving’ paradigm stimulated by the New Poor Law (see e.g. Lynch, 2016; King, 2019; Sims-Schouten, 2020). Between these positions was a tentative construction of ‘resilience’, with a focus on particular kinds of child who showed strength of character and an ability to change (Lynch, 2014; Parr, 1982). Yet other coping mechanisms, such as resistance to emigration and running away, were blamed on their upbringing and dismissed as difficult behaviour, classifying the child as hard to manage and undeserving (Moss, Wildman, and Lamont, 2020; Sims-Schouten, 2020; Sims-Schouten, Skinner, and Rivett, 2019). This article takes a closer look at child migration schemes, in order to paint a more complex picture of its practitioners, their perceptions of which children/young people should be included or excluded from the schemes, and the lives of children sent to the ‘land of opportunity’. Analysing archival materials from Canada alongside those from the UK offers insight into emigration decisions and justifications from philanthropic institutions in the UK and correspondence from children and parents prior to and after emigration to Canada. This is important in providing a more balanced overview of the range of perceptions and justifications tied to the child migration schemes, from those referring to the programmes as ‘child rescue schemes’ to those condemning child migrants as degenerate ‘slum kids’.

## ‘It would be a good opening for him’ and ‘One of the finest party of lads that has come to the city’

In the late 1800s, child migration to Canada was largely presented as an appropriate method for managing the large numbers of unsocialised, undisciplined, and neglected children taken on by the various philanthropic institutions. This was further justified by drawing attention to the need to protect such children from ‘immoral’ parents or other family members by despatching them to new homes and new lives overseas, sometimes without parental knowledge, let alone consent (Coldrey, 1999; Constantine, 1991, 2002). For example, the Waifs and Strays Society referred to the poor areas of London as constituting ‘a terrible pollution to the stream of our national life’ (Swain and Hillel, 2010: 67), in which children would be ‘contaminated from the outset by vicious surroundings’ (ibid.: 72). This moral framing of child redemption in the operation of these schemes existed alongside economic judgements – those viewing children as sources of cheap farm labour and domestic help – in complex and often contradictory ways (Lynch, 2016; Parr, 1982).

The Waifs and Strays Society started to send children to Canada from 1883 onwards. Below is an example of this, which relates to two brothers, one born in 1878 and the other in 1882; the application to the Waifs and Strays Society was made in 1893. The boys had been born in India, and the mother was described as ‘a lady of intemperate habits’. The father had abandoned the family. A letter from 1895 (an exact copy of which can be found in the case files of both boys) stated:

As this lad is anxious to go to Canada, & you think it would be a good opening for him it seems a pity for him not to do so. We have heard nothing now of the mother, & I should say we should be scarcely likely to do so now.

We have no objection to his going.^2^

By locating an element of desire – the wish to go to Canada – with the children themselves, this letter placed causality and the driving force behind the child migration scheme with the child, who was so ‘anxious to go’. Moreover, the construction of the absent mother, who was said to have shown little interest in the boys, provided a further incentive, and also shows that parental consent was not necessarily a priority. The child migration schemes, although praised for providing pauper children with a new life in the land of opportunity, were also subject to scrutiny. In 1875, for example, Andrew Doyle, a poor law inspector, carried out an investigation sanctioned by the London Board of Governors, who were tasked with overseeing child migration (to an extent, as most of this was left to the philanthropic agencies). In his report, which was largely disregarded, Doyle raised concerns about financial profiteering (by the voluntary agencies), as well as the fact that there were few or no regular follow-up visits with either the children or the families in which they had been placed (Bean and Melville, 1989; Kohli, 2003). Thus, there is a sense that, by foregrounding the desire of the child to be part of this scheme, the 1895 letter was attempting to silence any potential criticisms. Another example of correspondence, also from the 1890s, noted: ‘One of our boys in the Rochdale Home wishes to go to Canada next Spring. What arrangements must we make? ought he to go to one of our Farm Homes for a little training?’.^3^ As well as foregrounding the child's desire to go to Canada, this letter also suggested that the child would be of benefit to the receiving Homes in Canada, hence the reference to ‘training’. Moreover, tales of children doing well as a result of the schemes were also frequently used: ‘Another boy, W is from a Fegan's Home for Boys & he is now in Canada, near Ontario, & promised to do well there.’^4^

At the same time, while they initially welcomed the British children with open arms, as the children's schemes progressed Canadians became mistrusting of Britain’s intention to rid itself of the lowest of the low: idiots, the ill, and children with criminal intent (Bagnell, 2001; Parr, 1994). Such children, it was claimed, were not a valuable resource but ‘gutter snipes’, ‘often tainted with a hereditary disposition toward crime and viciousness’, such remarks hinting at the influence of biological determinism (Garland, 2018; Independent Inquiry Into Child Sexual Abuse, 2018). One item of correspondence from the Waifs and Strays Society in London referred to a girl, born in 1873, who was taken on by the Society in 1886. Her parents were separated, her father had died, and her mother had been found begging on the street. A letter from 1888 referred to the possibility of emigration to Canada, but this was eventually dismissed, because she was ‘disobedient and untruthful’, ‘inherently idle’, and ‘not quite all there it seems’.^5^

While some children on the surface appeared to adjust and showed ‘strength of character’ in light of the challenges they faced, other children expressed resistance to emigration by being hard to manage (Moss *et al.*, 2017; Moss, Wildman, and Lamont, 2020). It could be argued that the ‘disobedient and untruthful’ behaviour of the girl in the example above falls into the latter category and could be seen as a strategy of resistance in light of her having few means and methods of recourse. Yet the workings of biological determinism and the eugenic movement can also be seen here, in the description of her as ‘not quite there it seems’, as well as in decisions regarding which children should be allowed to go and which should be rejected or even sent back, such as ‘troublesome children’ and ‘bedwetters’.^6^ It could be argued that within the child migration schemes, children were a focal point of eugenic theory and practice, in terms of being constructed either as in need of protection and cultivation, or as problematic and imperfect and as such in need of being contained (Swain and Hillel, 2010; Wright, 2017). Stimulated by moral framing and biological determinism, the child migration selection procedures distinguished ‘innocent’ children with hope of a future from children for whom that innocence was complicated. The latter were constructed as a threat to innocence, as exhibiting undesirable symptoms of social and physical degeneracy (‘juvenile delinquent’ or ‘mentally deficient’), and as in need of being managed and controlled (Barham, 1999). In this case, this meant exclusion from the child migration schemes. For example, correspondence from a Waifs and Strays receiving home in Canada dated 1911 referred to ‘a boy … who has been in Canada nearly 8 years – emigrated from Lambeth workhouse – is said to be deficient and the Canadian authorities wish to send him back’.^7^ In the early 1900s, as eugenic ideas and fears regarding the genetic threat of feeble-mindedness became more prevalent, perceptions of defect and disability influenced the decision-making process, specifically in relation to which children/young people should be allowed to stay or should be sent back (Baker, 2014; Baynton, 2016).

In the early 1900s, there was a growing understanding of the multiple factors involved in child development, although the emphasis was predominantly on heredity (Rey *et al.*, 2015; Sims-Schouten, 2020). One of the most prominent movements applying genetics to the understanding of social and personality traits and related behaviour was the eugenics movement, established in the late 19th century (Garland, 2018). The eugenics movement, rooted in the biological determinist ideas of Sir Francis Galton, started to gain momentum from the 1880s onwards (Buss, 1976). G. Stanley Hall, an American psychologist with a specific interest in child development and eugenics, made studying children a priority in science (Partridge, 1912; Stewart, 2009). Hall's book on adolescence, published in 1904, was widely read across the Western world, including in Britain, and drew attention to the role of heredity and environment in moral development and psychopathology in childhood. For example, Hall made a link between ‘degenerate children’ and children who experienced fluctuating moods, showed aberrant tendencies under stress, or were sexually perverted or extremely shy. Moreover, he linked poverty to starvation of body and mind, leading to delays in the development and modification of physical structures and psychic powers (Hall, 1904). Correspondence from the Waifs and Strays Society also reflects this, referring to ‘hereditary pauperism and the Australian and USA Boarding out Schemes’.^8^

When UK child migration schemes to Canada were justified in the face of public opposition to these schemes in Canada itself, the moral framing of supplying Canada with ‘honest’ and ‘industrious’ youth and the notion that certain children were capable of redemption were often used to silence the critics (Lynch, 2014). This also fits with Smiles’ (1859; 1871) references in his books *Self-Help* and *Character* to self-control and character, both terms that are used in current research around resilience in children (e.g. Luthar, Cicchetti, and Becker, 2000; Sims-Schouten and Edwards, 2016). For example, correspondence from 1894 from the Waifs and Strays Society in relation to the possible emigration of a boy, aged 14 years old, to Canada stated, ‘His conduct is very good’ and ‘He has sound intellect’.^9^ Similarly, for some there was no redemption or hope of being sent to Canada. One case file from the Waifs and Strays Society refers to a girl, born in 1885, who was taken into care in 1889, aged 4. The application to the Waifs and Strays Society (in 1889) referred to this being a case of incest: ‘This is a very sad case, as the father of this child and her brother, is the mother's own father. She has had 5 children by him.’ Due to this unfortunate event, and despite the fact that ‘she is said to be a good girl, willing to work and well-conducted’, it was decided in 1900 that ‘this girl will not be suitable for emigration to Canada’. The reasons given were that ‘she is painfully slow and stupid, but I considered her mentally deficient’ and that ‘owing to her shameful birth, of which thank God, she is ignorant, she is weak in body and in mind, but she has been carefully and lovingly brought up and is truthful, gentle and God fearing’.^10^ This language describing the victim of incest also needs to be seen in light of eugenicist thinking and fears of race suicide more generally, specifically in relation to one of the earlier motives of the child migration schemes, which was to maintain the racial unity of the British Empire (Grier, 2002; Lynch, 2016). Seen in this light, the incest described above was a threat, and her being ‘painfully stupid’ and ‘mentally deficient’ were directly related to this and informed the decision to exclude her from the migration scheme over and above her good nature and careful upbringing and the moral motive of rescuing the child.

The complex relationship between economic judgements and moral framing in relation to the child migration schemes is also evident from the fact that in some situations the decision was made for one child within a family to be sent to Canada, while a sibling of that child was not deemed suitable. In such cases, there did not seem to be much regard for sibling relationships. An application letter to the Waifs and Strays Society dated 1893 referred to a girl, born in 1885, and asked for the girl to be taken away from her ‘wretched home’ due to poverty and bad treatment (her mother was dead and her father was described as ‘cruel’, and there was a reference to the fact that her brother was allowed to strike her).^11^ Another letter dated 1893, this time from the Grange in Uxbridge to Edward Rudolph, noted that ‘the poor child has had a sad history a life, but I think she will be very happy here’.^12^ In 1900, the girl was returned to the Waifs and Strays Society by the home: ‘She appears to be somewhat troublesome, being both untruthful and dishonest.’^13^ The case summary and application to the Society in 1893 also noted the following:

A year ago, not very long after his wife's death, I had the assistance of the Society for preventing Cruelty to Children, in consequence of his harshness, & neglect of his children. The consequence was that he was fined, along with his oldest daughter, – & intimation was given by the magistrate that if he was again convicted he wd. go to prison. He Home was wretched. Mr.XX took (by order of the magistrate) two of the children, S. & H., away entirely, & he has placed them in Homes. The oldest girl was turned out of doors by her father: one of my daughters got hold of her, & we sent her to a Training Home. She is now in respectable service but not earning wages. Another boy, W. was in the Fegan's Home for Boys: & he is now in Canada, near Ontario, & promised to do well there.^14^

By the early 1920s, the practice of allowing English children to be sent to Canada as, in effect, workers when child labour was no longer acceptable in England was being criticised, and the state reluctantly redrew the line between public welfare services and private charity (Constantine, 2002; Hammerton, 2017). This coincided with increased hostility from trade unionists and so-called child care specialists in Canada, who, touched by eugenicist ‘thinking’, continued to condemn child migrants as degenerate ‘slum kids’ (Constantine, 1991). Now, even more so than before, there is a sense of justifying the child's good nature, character, and intellect in the correspondence linked to children sent to Canada. Records from 1920–39 of receiving Fegan homes in Canada were mostly centred around character descriptions of the young boys sent from Britain, and also contained references to the boys’ usefulness in terms of work, again highlighting the complex relationship between economic judgements and moral framings within the child migration schemes.^15^ For example, a letter dated April 1925 referred to a boy admitted to a Fegan home in Canada in May 1920, aged 15, with specific reference to his character: ‘Character: A strong industrious boy, a bit on the heavy side temperamentally, but is not in any way mentally defective. Gets on with his job well without supervision not so quick in his uptake as some, but is a respectable, obedient helpful boy.’^16^ A boy admitted to a Fegan home in November 1922, aged 11, was described as having

rather a peculiar temperament – and is inclined to be sulky, but he soon gets over any fit of this kind – when he is understood he makes a fine worker. It would be well to put him with a man who can keep him up to the mark and he should prove very helpful. He is a greatly improved boy, since he came to us.^17^

Thus, sending and receiving homes had a tricky tightrope to walk: they were keen to send their paupered children to Canada, but at the same time they were keen to protect their reputation and send out good and industrious children. For example, correspondence from a Waifs and Strays receiving home dated 1929 referred to ‘one of the finest parties of lads that has come to the city the appearance and bearing of most of the lads was most favourably commented upon, and their educational standing is worthy of note’.^18^ Similarly, a Fegan receiving home referred to a boy, aged 14 and a half, who was admitted in June 1922 as ‘a really fine boy in body, mind and moral character – shy at first.… Health: Very good.… Stamina: Fine, healthy, bright boy’.^19^ This went both ways, however, and sometimes Canada itself was seen as a potential source of evil and temptations: ‘Toronto is not a good place for the girls to be in during their summer holidays’; ‘Many temptations’.^20^

Children sent to Canada arrived at receiving homes, from where they were distributed mainly to farms in need of young workers, for example in rural Ontario; few were adopted. Most were given bed and board and, as they got older, some wages, and many children ‘made good’, in the sense that they benefitted from jobs and modest living standards. Few did well, and many experienced abuse, in all forms (Independent Inquiry Into Child Sexual Abuse, 2018). The great majority suffered from trauma, firstly, from their disadvantaged (or worse) backgrounds in the UK, then from the separation from everything familiar when they were shipped overseas, and next from difficulties of all sorts that they endured in rural Canada (climate, hard physical labour, loneliness, lost identities, living with a family but not being part of the family, etc.; Constantine, 2002; Kohli, 2003; Lynch, 2016). The next section explores the specific and ascribed traits, characteristics, strengths, and weaknesses of those children who did and did not do well following emigration to Canada, addressing issues around resistance, character, courage, and self-control (Moss, Wildman, and Lamont, 2020; Smiles, 1859, 1871). While these qualities are currently referred to as ‘resilience’, which represents the ability to rebound from acute or chronic adversity (Vernon, 2004), there is as yet no consensus on the referent of the term, the standards for its application, or its role in explanation, models, and theories (Glantz and Slobada, 1999; Ungar, 2004).

## ‘Happy’, ‘doing very well’, and ‘good character’ versus ‘sensitivities’ and ‘not strong enough’

Down through the decades, theorists, professionals, and politicians have set the stage for various viewpoints regarding child protection and support practices, with the period of the late 19th to the early 20th century being one of significant reform in ideas and practices pertaining to children (Cradock, 2014; Hendrick, 1997; Sims-Schouten, 2020). With this came a focus on three distinct forms of ‘normal’ childhood, namely normal as healthy, as average, and as acceptable. Here, ‘normal’ was contextualised and legitimated by measuring it against the ‘abnormal’. The latter was initially associated with physical traits but became increasingly synonymous with perceived deficits in mental capacity, personality, and conduct (Wright, 2017). Inherent in the construction of ‘abnormality’ was a focus on personalities lacking the capacity to change and incapable of redemption. The specific groups seen as irredeemable varied across the different contexts in which this moral frame was used, but as can be seen from the previous section, what was common across these different cases was the symbolic construction of them as ‘others’ – too morally polluted to be capable of being purified (Chauhan and Foster, 2014; Lynch, 2014; Roberts and Schiavenato, 2017). Such modes of classification were also located within wider eugenic concerns around biological determinism and social efficiency, with little regard for the fact that some of the behaviours in question could represent strategies of resistance by children who had few means and methods of recourse (Moss, Wildman, and Lamont, 2020; Wright, 2017). In contrast to this were the ‘normal’ and acceptable children, the ones who did well and whose behaviour and achievements were celebrated. For example, a letter from Edward Rudolf, the founder of the Waifs and Strays Society, written in May 1910 stated:

I have heard that the above-named boy, who was originally in the Standon Home, and left there in 1890 to enter the office at Headquarters, is now Mayor of a town in *Canada*. Do you happen to have a recent photograph of him, as if so I shall be glad if you will send it to me as I should like to make use of it in the Magazine.

Yours faithfully, E. de M. Rudolf^21^

The magazine referred to in the letter was *Our Waifs and Strays*, the quarterly paper of the Waifs and Strays Society, a newsletter for supporters and donators, first published in October 1882. The magazine reported on the positive stories and experiences of the Society. Children who did not fare so well tended not to be featured in the magazine. Correspondence linked to the latter category of children often attributed these children's failure to thrive and do well to ‘sensitivities’ or lack of strength of character. This was located within the pauper child and their poor constitution, family background, and troubled moral nature; for some, this meant that there was no hope of redemption or possibility of them being converted into good, serviceable citizens (King, 2019; Lynch, 2014). Here again, the link with biological determinism and inherited tendencies, both physical and moral, raised its head (Buss, 1976; Garland, 2018). The perceived civic and moral threat posed by these children was also grounded in the broader moral frame that these child welfare schemes drew on: that potentially redeemable children were at risk of becoming morally tainted through their prolonged exposure to particular kinds of social environment (Bean and Melville, 1989; Coldrey, 1999).

The framings of the child ‘capable of change’ and the ‘hard-to-manage child’, as well as judgements around ‘sensitivities’ and ‘strength of character’, can be analysed in light of Smiles’ publications *Self-Help* and *Character*, as well as resistance and resilience (Moss, Wildman, and Lamont, 2020; Ungar, 2002, 2004). Although the terms were not used in research and practice until the 1970s, the language surrounding the child migration schemes hints at engagement with key concepts that are now referred to as ‘resilience’: ‘positive traits’, ‘character’, and ‘adaptability’, to name a few (Luthar, Cicchetti, and Becker, 2000; Masten, 2014; Rutter, 1993). Werner, who undertook the first known study on ‘resilience’ in the 1970s, studied a group of children in Hawaii who lived in poverty and had parents who were alcoholics and had mental health problems. Werner and Smith (1977, 1982) found that two-thirds of the children growing up in these circumstances exhibited ‘destructive’ behaviour and one-third demonstrated more ‘positive’ traits; they called the latter group ‘resilient’. According to Werner and Smith (1977), the resilient children had particular individual characteristics, namely they were liked by peers and adults, they were reflective rather than impulsive, and they were able to use flexible coping strategies to overcome adversity. Since then, significant research has been undertaken with a focus on resilience (Masten, 2014; Rutter, 1993; Ungar, 2004), mostly with a focus on ‘positive emotions’, ‘successful traits’, and coping mechanisms that allow people to be more or less resilient in the face of adversity. There are currently several waves of resilience research and theory, each building on its predecessors: the first wave, focussing on the individual and descriptions of resilience and related methodologies; the second wave, adopting a developmental systems approach to theory and research; the third wave, focussing on interventions directed at changing developmental pathways; and the fourth wave, integrating multiple levels and systems (epigenetics, biology, and culture; Wright, Masten, and Narayan, 2013). Yet the general consensus is that resilience is marked by ‘strengths’ and ‘positive’ coping mechanisms and behaviours in light of adversity. It could be argued, however, that resilient young people take advantage of whatever opportunities and resources are available to them, even those considered, on the surface, negative or destructive (Sims-Schouten, 2020; Ungar, 2002). The latter could be seen as strategies of resistance in light of having few means and methods of recourse (Moss *et al.*, 2017; Moss, Wildman, and Lamont, 2020).

Correspondence and archives relating to the child migration schemes to Canada consist of many different layers, from initial tentative discussions around whether a child would be appropriate for migration to Canada, to chairman's reports on ‘cases for emigration’, including decisions (e.g. those candidates ‘passed’ or ‘deferred’ for various reasons, including those with physical ailments or disabilities and those who were ‘not strong enough’, ‘rather under age’, or ‘not to be trusted’) and reports and letters from the receiving home. Moreover, case files also hold letters from children; for example, a letter held in the LAC collection from a child indicates that the young person was not looking forward to travelling to Canada.^22^ Once children who were deemed acceptable to take part in the child migration schemes arrived in Canada, correspondence was sent from receiving homes providing brief updates on the young person's progress and character. Inspection letters can also be found in the archives. For example, correspondence from a receiving Fegan home in 1887 referred to ‘character from the Home: – Grant, *worth his weight in gold*’ in relation to a 12-year-old boy emigrated from England*.*^23^ Further correspondence regarding the boy, in September 1888, highlighted that he was ‘most satisfactory, a little *slow but* trustworthy and good’*.*^24^ Correspondence following a visit in March 1891 reported on the fact that the young man was ‘very much improved’ and ‘very happy and much lifted’. Another report dated shortly after this, in April 1891, noted: ‘Visited. Looking Well.… Happy and content.… Sore feet.’^25^ In May 1893, it was reported that he ‘takes his own money’.^26^ The last recorded visit was in 1896.^27^ Letters from receiving homes also referred to general progress, as well as issues such as ‘the boy has disappeared’, which appears in the Waifs and Strays files from 1916.^28^ In addition to this, some reports and chairman’s minutes make reference of children being returned to England. Correspondence from the Waifs and Strays Society in 1914 also mentioned that ‘girls cannot be returned’.^29^ It is not clear why ‘girls cannot be returned’, but a Juvenile Deportations List compiled at LAC consisting of information surrounding deportations between 1910 and 1933 reveals that only a fraction of girls, compared to boys, were returned to the UK: of the 823 deportations on the list, 62 are girls and 761 are boys.^30^

In some cases, initial correspondence regarding the young person's good character, trustworthiness, and satisfactory work ethic was followed by reports of suicide. In these cases, it was the young person's ‘unsound mind’ or ‘sensitivities’ that were held accountable. The Waifs and Strays Society archives in London contain a number of case files where references were made to suicide after the young person had been sent to Canada. All of these related to boys, and for all of them this marked the end of the correspondence; there was no follow-up or further communication. For example, one case file reported on a young boy, born in 1915, who was sent to Canada in 1930. The final correspondence in relation to this young man appeared in 1935, reporting that he had committed suicide by hanging himself from a beam in a barn at his workplace. It was assumed a ‘mental aberration occurred, following upon an obsession for detective stories’.^31^ Another case file from the Society archives referred to a boy, born in 1909, who was taken on by the Society in 1919. There was talk of him coming from a wretched family and a note that no visits from his father were allowed. In 1927, he was sent to Canada, and his application affirmed that ‘the lad bears a good character’. The final item of correspondence in 1933 noted that he had ‘committed suicide by shooting himself’.^32^ A different case file, again from the Society archives, concerned a boy born in 1910; his application to the Society was made in 1923, when the boy was 13 years old. The application stated that the child had been deserted by his father and that his mother was dead. In 1927, he was sent to Canada, and a final letter from Canada reported that he had ‘committed suicide by hanging’; the letter also referred to his ‘sensitivities’.^33^

‘Self-help’, ‘character’, and ‘resilience’ concern the ability to ‘bounce back’ and relate to doing well against the odds, coping, and recovering (Rutter, 1993; Smiles, 1859, 1871; Stein, 2006). Samuel Smiles promoted courage, self-control, thrift, and responsible habits in his books *Self-Help* and *Character*, while *resilience* is defined in terms of a process of and capacity for successful adaptation despite challenging or threatening circumstances (Masten, Best, and Garmezy, 1990). In relation to the child migration schemes, the moral framing of ‘saving the child from pauperism’ was in stark contrast to the way in which the child was held accountable for many things once they had arrived in Canada, including suicide, which was constructed in terms of weaknesses within the child. Only a few cases referred to bad treatment in Canada: a Fegan Homes file, for example, referred to ‘cruel behaviour of master’, while the Waifs and Strays Society files also contain some letters from children complaining about bad treatment in the Waifs and Strays homes.^34^ On some occasions this was investigated, but in most situations it was concluded that the child had made up bad stories, was an attention seeker, or was simply wrong, as happened in the above case*.* Discussions of resilience, self-help, and character are typically framed with reference to risk, vulnerability, and protective factors (Smiles, 1871; Ungar, 2005; Werner and Smith, 1982). Yet in narratives and perceptions around ‘troubled children’, as can be seen from the analysis above, it was their behaviour that was being judged, and their capacity to develop resilience or resistance within this was not recognised (Moss *et al.*, 2017; Moss, Wildman, and Lamont, 2020; Sims-Schouten and Riley, 2018; Sims-Schouten, Skinner, and Rivett, 2019).

## Discussion

This article has engaged in a critical reflection on the ideals and rationales of Canadian child emigration schemes, associated with Fegan Homes and the Waifs and Strays Society. Specifically, it has been shown how these schemes were presented as moral programmes to rescue children facing poverty or danger, while at the same time they adopted discriminatory selection procedures framed within stereotypical judgements regarding ‘bad behaviour’ and mental inferiority. Within this, the ‘rescued’ child or young person was positioned within a lower class/hierarchy and stigmatised as less worthy or able than other children (Sohasky, 2015). Moreover, decisions around child migration were fuelled by deterministic assumptions associated with biological determinism, namely that a child's moral character was irretrievably shaped by heredity, condemning child migrants as degenerate ‘slum kids’. At the same time, reference was made to the young person's ‘desire’ to go to Canada, and the philanthropic organisations dedicated a considerable amount of correspondence to the benefits of the scheme for both the child and the receiving home in Canada, as well as the child's ‘character’ and ‘ability to cope/adjust’. Mentions of the latter both fit within Samuel Smiles’ discussion of the influence of character, courage, self-control, home power, and temper in his books Self*-Help* and *Character*, and can be viewed as early references to ‘resilience’, the ability to cope in the face of adversity (Moss, Wildman, and Lamont, 2020; Ungar, 2002, 2004). Yet this ability was referred to in terms of the child's strengths, with little regard for other factors or the definition of *strength*. However, in light of the traumatic experiences that the children may have been exposed to, strengths could also lie in ‘disordered’ or delinquent behaviour, which could be viewed as a strategy of resistance by children who had few means and methods of recourse (Moss, Wildman, and Lamont, 2020; Ungar, 2002, 2004). As Moss, Wildman, and Lamont (2020) argue, while some children, on the surface, appeared to adjust and showed ‘strength of character’ in light of the challenges they faced, other children expressed resistance to emigration by being hard to manage.

Thus, it could be argued that resilient young people take advantage of whatever opportunities and resources are available to them, even those considered, on the surface, negative or destructive (Sims-Schouten, 2020; Ungar, 2002). As a result, negative behaviour seen in troubled young people can actually signal a pathway to resistance, a form of hidden resilience that is, just like those chosen by their well-behaved peers, simply focussed on the need to create powerful and influential identities for themselves. Yet it is those children who are generally perceived as ‘troublesome’, as well as ‘disobedient and untruthful’. ‘Resilient’ and ‘capable’ children, on the other hand, represent the group of children who manage to cope with uncertainty and are able to recover successfully from trauma (Masten, 2014). The period of the late 19th to the early 20th century was one of significant reform in ideas and practices pertaining to children, with ‘normal’ childhood being described as ‘healthy’, ‘average’, and ‘acceptable’ (Cradock, 2014; Hendrick, 1997; Sims-Schouten, 2020). Moreover, the ‘normal’ was contextualised and legitimated by being measured against the ‘abnormal’, which became increasingly synonymous with perceived deficits in mental capacity, personality, and conduct (Wright, 2017). Similar conceptualisations can be seen in modern discussions of the ‘resilient’ child, who is generally referred to in terms of the seven crucial *C*'s: competence, confidence, connection, character, contribution, coping, and control (Luthar, Cicchetti, and Becker, 2000). Here, responsibility appears to be located largely within the young person and their ‘internal assets’ (mental capacity, personality, and conduct), the premise being that children and young people who have healthy strategies in place may be less likely to turn to troublesome or ‘bad’ behaviour to relieve stress (Lösel and Bender, 2003; Masten, Best, and Garmezy, 1990). At the same time, when things go wrong for children previously described as having ‘good character’ and ‘doing very well’, this is also located within the child and flaws in their ability to cope. This can be seen from the reference to ‘not strong enough’ in the correspondence relating to children who were excluded from the child migration schemes and ‘sensitivities’ in relation to those who committed suicide.

Notions to do with ‘morality’ and ‘behaviour’ are highly influential in past and present conceptualisations of childcare and child protection (bearing in mind that current social work and social care practice were also born of the child rescue movement that gave rise to the child migration schemes in the 1800s) and are often used to refer to a relationship of mind, body, and social environment (Fong *et al.*, 2018; Jones *et al.*, 2018; Sohasky, 2015). The focus here is largely on a reductionist or isolated notion of the individual, who is blamed for their ‘bad’ behaviour and ‘inherited tendencies’, rather than on large-scale social structures (Dagnan, 2007; Toms, 2012). In practice, this translates into assessments and interventions at various levels, from individual experiences and behaviour through to dynamics in the immediate social context, essentially locating ‘problems’ within the child and their family background (think about the child who was excluded from the child migration scheme due to incest in the family; Chettiar, 2012; Sinhg and Tuomainen, 2015; Slack and Webber, 2008). The tenets of critical realism encourage a focus on the interaction between structure and agency in stratified entities, viewing context or situational influences as crucial to an understanding of processes and emergent outcomes (Kessler and Bach, 2014; Saka-Helmhout, 2014). As a result, critical realism is instrumental in influencing the search for generative mechanisms that may have combined to create a phenomenon over time, influencing particular outcomes and practices (Sims-Schouten, 2020; Sims-Schouten, Skinner, and Rivett, 2019).

The archival material (correspondence, case files, emigration paperwork, reports, and magazines) drawn upon in this article illuminates both broader cultural frameworks and constraints within this and significant mechanisms that provide situational logics for action/inaction (Mutch, 2014). For example, the moral framing of child redemption in the operation of the child migration schemes existed alongside economic judgements – such as the conception of children as sources of cheap farm labour and domestic help – in complex and often contradictory ways (Lynch, 2016; Parr, 1982). This was further complicated by the intricate interplay between morality, biological determinism, and resilience in decisions around which children should be included/excluded. This meant that children were subjected to judgements about their mental ability and related behaviour, with little regard for their early experiences of abuse and neglect, or for the fact that many suffered abuse in the land of opportunity. Instead, the focus was on individual accountability and responsibility, which strongly resembled the ‘deserving/undeserving’ paradigm promoted by the New Poor Law and the harsh related philosophy of self-care and self-responsibility (Sims-Schouten, Skinner, and Rivett, 2019; Skinner and Thomas, 2018). In judgements around ‘deservedness’, related stigmas around poverty and ‘bad’ behaviour were rife. Within this, the child was punished for his/her ‘immoral tendencies’ and ‘inherited traits’, with little regard for the underlying reasons (e.g. abuse and neglect) for their (abnormal) behaviour and ‘mental deficiencies’ (Fisher *et al.*, 2000; Hardwick, 2005). It was the complex interplay and nuance between conceptions of the moral/immoral, desirable/undesirable, degenerate, and capable/incapable child that guided practice with vulnerable children in the late 1800s, a legacy that can still be seen in social care decisions today, highlighting the need for greater reflection on conceptualisations and ‘realities’ of ‘problem children’ and the pathways to resilience inherent in this (Sims-Schouten, 2020; Ungar, 2002, 2004, 2005).

## Notes

1. Children’s Society archives, available at *Hidden Lives Revisited* (https://www.hiddenlives.org.uk) and *The Children’s Society* (http://www.calmview.eu/ChildrenSociety/CalmView/default.aspx).

2. Letter from a Waifs and Strays receiving home addressed to Edward Rudolph about emigration to Canada, 1895, Waifs and Strays Society Archives, London (hereafter ‘W&S Archives’), Cases 3622/3623.

3. Letter from Rochdale Home addressed to Edward Rudolph about emigration to Canada, 1899, W&S Archives, Case 3967.

4. Case file, 1893, W&S Archives, Case 3737. This is a case file relating to a girl who was taken into care in 1893, aged 8 years old. In the case file is a letter from Edward Rudolph referring to her brother, who had been sent to Canada and was doing well.

5. Letter between a Waifs and Strays home and Edward Rudolph in relation to emigration to Canada, 1888, W&S Archives, Case 718.

6. Library and Archives Canada, Waifs and Strays Archives (hereafter ‘LAC W&S’), Homes Abroad and Emigration Papers (Sherbrooke, Quebec), 1905–20, A-1138 to A-1139, 1893.

7. LAC W&S, Homes Abroad and Emigration Papers (Sherbrooke, Quebec), 1905–20, A-1139, 1911.

8. LAC W&S, A-1141 to A-1142, 1882–9 (bound volumes of printed papers entitled *Our Waifs and Strays*).

9. Letter to Edward Rudolph in relation to emigration to Canada, 1894, W&S Archives, Case 572.

10. Application to Waifs and Strays Society, 1889, W&S Archives, Case 2274; and Correspondence between Edward Rudolph and a children's home about emigration to Canada, 1900, W&S Archives, Case 2274.

11. Application letter to Waifs and Strays Society, 1893, W&S Archives, Case 3737.

12. Letter from the Grange in Uxbridge to Edward Rudolph, 1893, W&S Archives, Case 3737.

13. Letter from the Grange to Rudolph, 1900.

14. Case summary and application to Waifs and Strays Society, 1893, W&S Archives, Case 3737.

15. Library and Archives Canada, Fegan Homes Archives (hereafter ‘LAC FHA’), Vols 7 and 8 (records of receiving Fegan Homes in Canada, 1920–39).

16. Correspondence from a receiving Fegan Home, 1925, LAC FHA, Vol. 7.

17. Correspondence from a receiving Fegan Home, 1922, LAC FHA, Vol. 7.

18. Register Emigration Parties book, Sherbrooke Record, 1929, LAC W&S, A-1137.

19. Correspondence from a receiving Fegan Home, 1922, LAC FHA, Vol. 7.

20. LAC W&S, Annual Reports, 1882–96, A-1140, 1896.

21. Letter from Edward Rudolph to a receiving Home in Canada, 1910, W&S Archives, Case 512.

22. Letter from a young person to the Lord Bishop of London, 1929, LAC W&S, A-1139.

23. Correspondence from a receiving Fegan Home, 1887, LAC FHA, Vol. 2 (emphasis in original).

24. Correspondence from a receiving Fegan Home, 1888, LAC FHA, Vol. 2 (emphasis in original).

25. Correspondence from a receiving Fegan Home, 1891, LAC FHA, Vol. 2.

26. Correspondence from a receiving Fegan Home, 1893, LAC FHA, Vol. 2.

27. Correspondence from a receiving Fegan Home, 1896, LAC FHA, Vol. 2.

28. Chairman's notes, 1916, LAC W&S, A-1137.

29. Chairman’s notes, 1914, LAC W&S, A-1137.

30. Library and Archives Canada, Juvenile Deportations and Charity, 18183, 1910–33.

31. Correspondence and application in relation to a young boy and emigration to Canada, and subsequent suicide, 1915–35, W&S Archives, Case 20244.

32. Correspondence and application in relation to a young boy and emigration to Canada, and subsequent suicide, 1919–31, W&S Archives, Case 23900.

33. Correspondence and application in relation to a young boy and emigration to Canada, and subsequent suicide, 1923–31, W&S Archives, Case 29107.

34. Correspondence from a receiving Home, 1885, LAC FHA, Vol. 1; and Correspondence between a young boy and Edward Rudolph about bad treatment in a receiving home, 1901, W&S Archives, Case 8645 (the accusation was eventually dismissed as untrue).
